# The utility of gadoxetic acid-enhanced magnetic resonance imaging in the surveillance for postoperative recurrence of hepatocellular carcinoma

**DOI:** 10.1097/MD.0000000000005666

**Published:** 2016-12-23

**Authors:** Jung Hee Kim, Yang Won Min, Geum-Youn Gwak, Yong Han Paik, Moon Seok Choi, Joon Hyoek Lee, Kwang Cheol Koh, Seung Woon Paik

**Affiliations:** Department of Medicine, Samsung Medical Center, Sungkyunkwan University School of Medicine, Seoul, Korea.

**Keywords:** enhanced MRI, gadoxetic acid, hepatic resection, hepatocellular carcinoma, surveillance

## Abstract

This study aimed to investigate the utility of gadoxetic acid-enhanced magnetic resonance imaging (Gd-MRI) in surveillance for recurrent hepatocellular carcinoma (HCC) after hepatectomy.

This retrospective study analyzed 147 patients who underwent surveillance with alternating multidetector computed tomography (MDCT) and Gd-MRI after hepatectomy for HCC. The patients were followed-up every 3 months during the first 2 years, and every 6 months thereafter. At each visit, MDCT was performed but once a year (every 12 months), Gd-MRI was performed instead of MDCT. Each HCC recurrence detection rate of MDCT and Gd-MRI was evaluated, and recurrent HCC characteristics were compared according to the detection test.

A total of 63 patients had recurrent HCC. Among them, 9 were detected with Gd-MRI and 29 with MDCT. The baseline characteristics of patients with recurrent HCC showed no significant differences according to the detection test. The HCC recurrence detection rate of Gd-MRI and MDCT was 4.8% (9/180) and 4.3% (29/580), respectively, on the per test basis (*P* = 0.764). However, in the population with a follow-up period of ≥12 months, the detection rate of Gd-MRI and MDCT was 4.3% (7/150) and 1.5% (19/400), respectively (*P* = 0.035). Recurrent HCCs detected with Gd-MRI were smaller than those detected with MDCT (tumor size < 2 cm, 100% vs 65.5%, *P* = 0.040).

Our data suggest that Gd-MRI has advantages in detecting recurrent HCC after hepatectomy. Surveillance with alternating MDCT and Gd-MRI may identify more recurrent HCC in an early stage than with MDCT alone in patients who received hepatectomy for HCC.

## 1 Introduction

Hepatocellular carcinoma (HCC) is the sixth most common neoplasm and the third most frequent cause of cancer death.^[1]^ Hepatic resection is the treatment of choice for HCC, although careful selection of candidates is vital to avoid treatment-related complications in individuals with cirrhosis.^[2]^ However, the tumor recurrence rate, including true recurrence arising within the first 2 years after resection and de novo tumors, is reported to exceed 70% at 5 years.^[3]^ Therefore, early detection for recurrent HCC is necessary to improve survival.^[4]^ It is generally accepted that the period of high risk for HCC recurrence is between 1 and 2 years after operation when most recurrent cases are detected by “periodic” imaging studies.^[5]^ A combined use of alpha-fetoprotein (AFP) and/or prothrombin induced by vitamin K absence II (PIVKA II) and imaging studies, such as ultrasonography (US), computed tomography (CT), and magnetic resonance imaging (MRI), is usually performed for postoperative surveillance. However, there is little data thus far to indicate superiority of one imaging modality over the other for surveillance of postoperative HCC recurrence.^[6]^ Furthermore, data are largely lacking in the ideal imaging interval.

The diagnostic imaging of HCC has recently undergone marked progress. The use of liver-specific contrast agents such as gadolinium ethoxybenzyl diethylentriamine pentaacetic acid (Gd-EOB-DTPA, Primovist, Bayer Schering Pharma, Berlin, Germany) produces both dynamic and liver-specific hepatobiliary MR images, thus improving both the detection and characterization of focal liver lesions.^[7,8]^ A few recent studies reported that gadoxetic acid-enhanced MRI (Gd-MRI) yielded superior diagnostic performance in HCC detection in comparison with multidetector CT (MDCT).^[9,10]^

Accordingly, we hypothesized that surveillance with alternating MDCT and Gd-MRI could have an advantage in detecting recurrent HCC after hepatectomy as compared with MDCT alone. The present study aimed to evaluate the surveillance performance of alternating MDCT and Gd-MRI in postoperative HCC patients.

## 2 Methods

### 2.1 Patient selection

**Figure 1:**
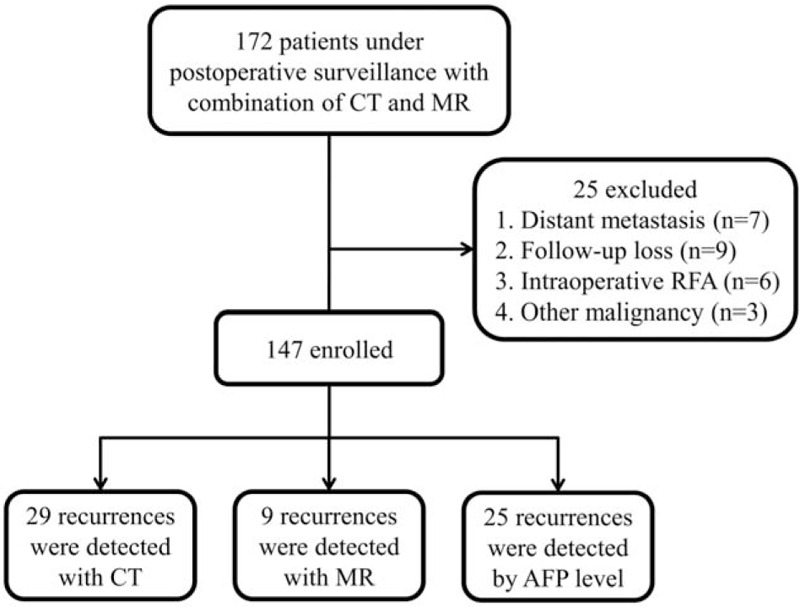
Flow chart. AFP = alpha-fetoprotein, CT = computed tomography, MR = magnetic resonance, RFA = radiofrequency ablation.

This retrospective cohort study was conducted according to the principles of the Declaration of Helsinki. The study involved HCC patients who underwent surveillance under one hepatologist (SW Paik) after hepatectomy for the first diagnosed HCC at Samsung Medical Center, Seoul, Korea during the period between January 2008 and December 2010. The strategy of postoperative surveillance for HCC patients was as follows. The patients were followed-up every 3 months during the first 2 years, and every 6 months thereafter. At each visit, liver function tests, serum AFP level measurements and MDCT were performed for surveillance of recurrence. Once a year (every 12 months), Gd-MRI was performed instead of MDCT. MDCT and Gd-MRI were not performed at the same time point. In other words, 4th follow-up MDCT was substituted by 1st follow-up Gd-MRI at 1 year. Patients who met any of the following criteria were excluded: subjects who received hepatic resection with intraoperative radiofrequency ablation (n = 6); subjects who were lost to follow-up within 12 months after hepatectomy (n = 9); subjects who were detected with extrahepatic metastasis during surveillance (n = 7); and history of any other cancer (n = 3). Additionally, subjects who underwent Gd-MRI with suspicion of recurrence from increasing AFP level were excluded from the analysis (n = 25). Finally, a total of 122 patients were enrolled (Fig. 1). The study was reviewed and approved by the Institutional Review Board at Samsung Medical Center.

### 2.2 Data collection

The following clinical and laboratory information was collected from each patient: age, gender, etiology of liver disease, platelet count, serum albumin and bilirubin levels, prothrombin time (PT), Child–Pugh class, serum AFP level, and the extent of hepatectomy. According to the pathological reports, the fibrosis stage of the background liver and Edmondson–Steiner classification and the modified UICC stage of cancer were determined.

### 2.3 Imaging methods

Multiphase (contrast-enhanced hepatic arterial, portal venous, and equilibrium phases) CT was conducted with a 40-MDCT scanner (Brilliance 40, Philips Healthcare, Highland Heights, OH, USA ) and with a 64-MDCT scanner (Aquilion 64, Toshiba Medical,Tochigi Prefecture, Japan and LightSpeed VCT 64, GE Healthcare Milwaukee, WI, USA). The scanning parameters were as follows: 120 kVp, 189–200 mAs, a slice thickness of 5-mm with an increment (overlap) of 2.5 mm, table speed of 26.5 to 39.37 mm/rotation (pitch, 0.828–1.07), and a single-breath-hold helical acquisition of 4 to 6 seconds depending on the liver size. Images were obtained in the craniocaudal direction. Hepatic arterial phase scanning began 30 to 40 seconds after injection of 120 mL of a nonionic iodinated contrast agent (Iopamidol, Iopamiro 300, Bracco) at a rate of 3 to 4 mL/second by means of a bolus-triggered technique (120 kVp; 40–60 mA; monitoring frequency from 12 seconds after the contrast injection, 1 second; trigger threshold, 100 HU in descending aorta; delay from trigger to initiation of scan, 18 seconds). The contrast agent was administered through the antecubital vein with a power injector. The portal and equilibrium phases of scanning began 70 and 180 seconds after injection of the contrast agent, respectively. MRI was conducted using a 3.0 T whole-body MRI system (Intera Achieva 3.0 T, Philips Healthcare) with a 16-channel phased-array coil as the receiver coil. For Gd-MRI, unenhanced, arterial phase (20–35 seconds), portal phase (60 seconds), late phase (3 minutes), and 20-minute delayed hepatobiliary phase images were obtained with a T1-weighted 3D turbo-field-echo sequence (T1 high-resolution isotropic volume examination, THRIVE, Philips Healthcare) with a 2-mm section thickness, and a field of view of 32 to 38 cm.

### 2.4 Assessment

The diagnosis of HCC including recurrence was based either on pathologic confirmation or on the clinical criteria.^[11,12]^ The clinical diagnosis of HCC was made when the AFP level was ≥200 ng/mL and at least one of the dynamic enhancement CT or MRI showed a vascular pattern typical of HCC in patients at risk including patients with HBV or HCV infection, or liver cirrhosis. If the AFP level was <200 ng/mL, at least 2 of the dynamic enhancement CT, MRI, or transarterial angiography must show vascular patterns typical of HCC in order to make a diagnosis of HCC. In patients with cirrhosis, the diagnosis of HCC was made when the tumor was same or larger than 2 cm and at least one of the dynamic enhancement CT or MRI showed a vascular pattern typical of HCC, irrespective of AFP level.

The primary outcome was the recurrent HCC detection rate, which was defined as the proportion of tests which detected recurrent HCC among the total performed tests. Each recurrent HCC detection rate of Gd-MRI and MDCT was evaluated. As a secondary outcome, the characteristics of the detected recurrent HCCs were evaluated in terms of tumor size, tumor number, and required additional treatment. In addition, the likelihood of recurrent HCC detection, which was recurrent HCC detection rate in the following surveillance after a negative result of either Gd-MRI or MDCT, was evaluated.

### 2.5 Statistical analysis

The statistical results are presented as mean ± SD, number (percentages) or median (range). Continuous variables were compared parametrically using Student *t* test or nonparametrically using the Mann–Whitney *U* test. Categorical variables were compared using the *χ*^2^ test or Fisher exact test as appropriate. Recurrent HCC detection rates were compared using the McNemar test. A 2-sided *P*-value < 0.05 was considered statistically significant.

## 3 Results

A total of 63 patients had recurrent HCC. Among them, 9 patients were detected with Gd-MRI, 29 patients with MDCT, and 25 patients who had increased AFP level were excluded from the analysis. The baseline characteristics of patients with recurrent HCC showed no significant differences according to the detection test, age, gender, etiology of liver disease, platelet count, serum albumin and bilirubin levels, PT, Child–Pugh class, serum AFP level, fibrosis stage, Edmondson–Steiner class, modified UICC stage, and hepatectomy extent (Table 1).

**Table 1:**
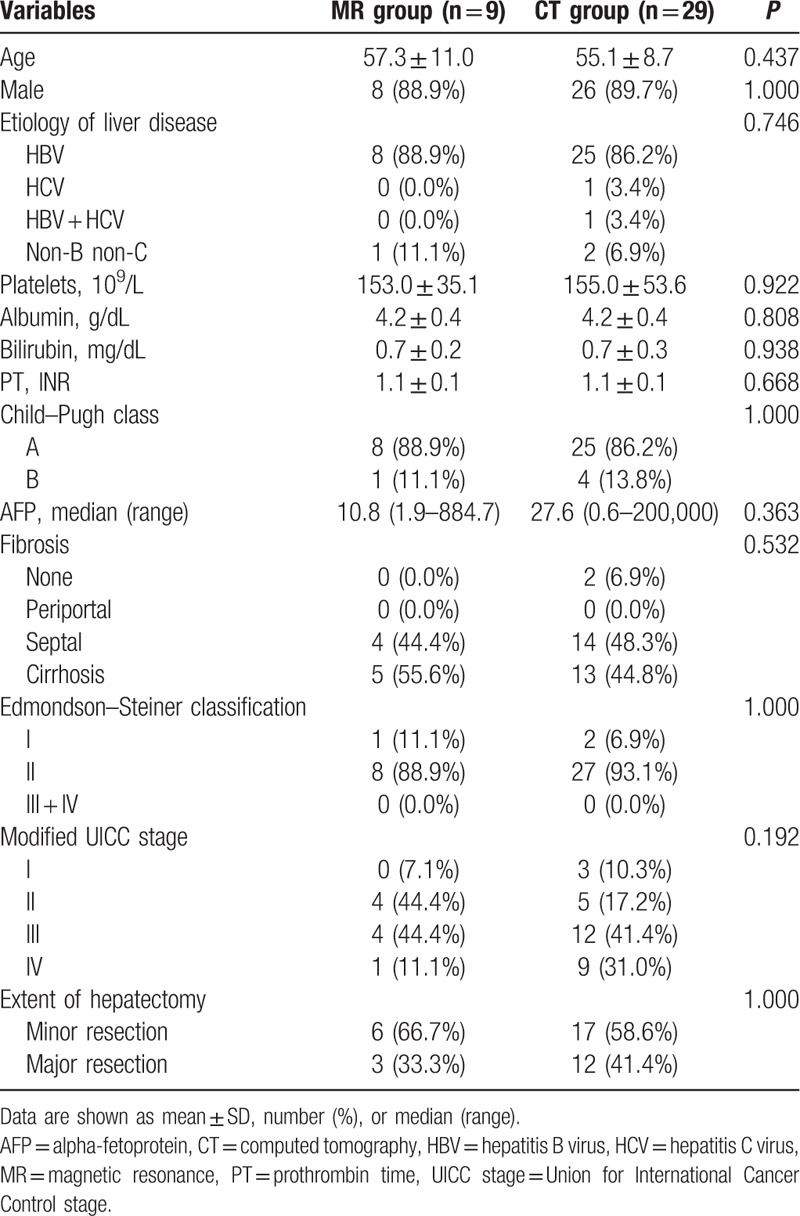
Comparison of baseline characteristics between recurrent hepatocellular carcinoma patients who were detected with godoxetic-enhanced magnetic resonance imaging (MRI) and those detected with multidetector computed tomography (MDCT).

### 3.1 Recurrent HCC detection rate

**Figure 2:**
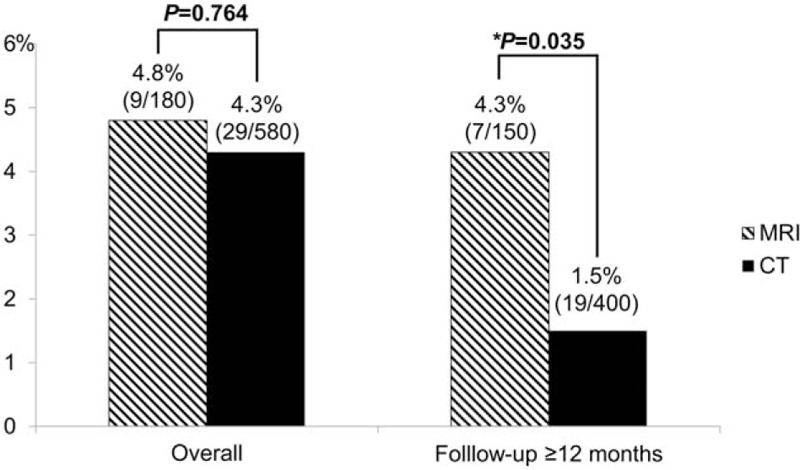
Comparison of recurrent hepatocellular carcinoma detection rates according to the surveillance test. CT = computed tomography, MRI = magnetic resonance imaging. ∗*P*-value <0.05.

**Figure 3:**
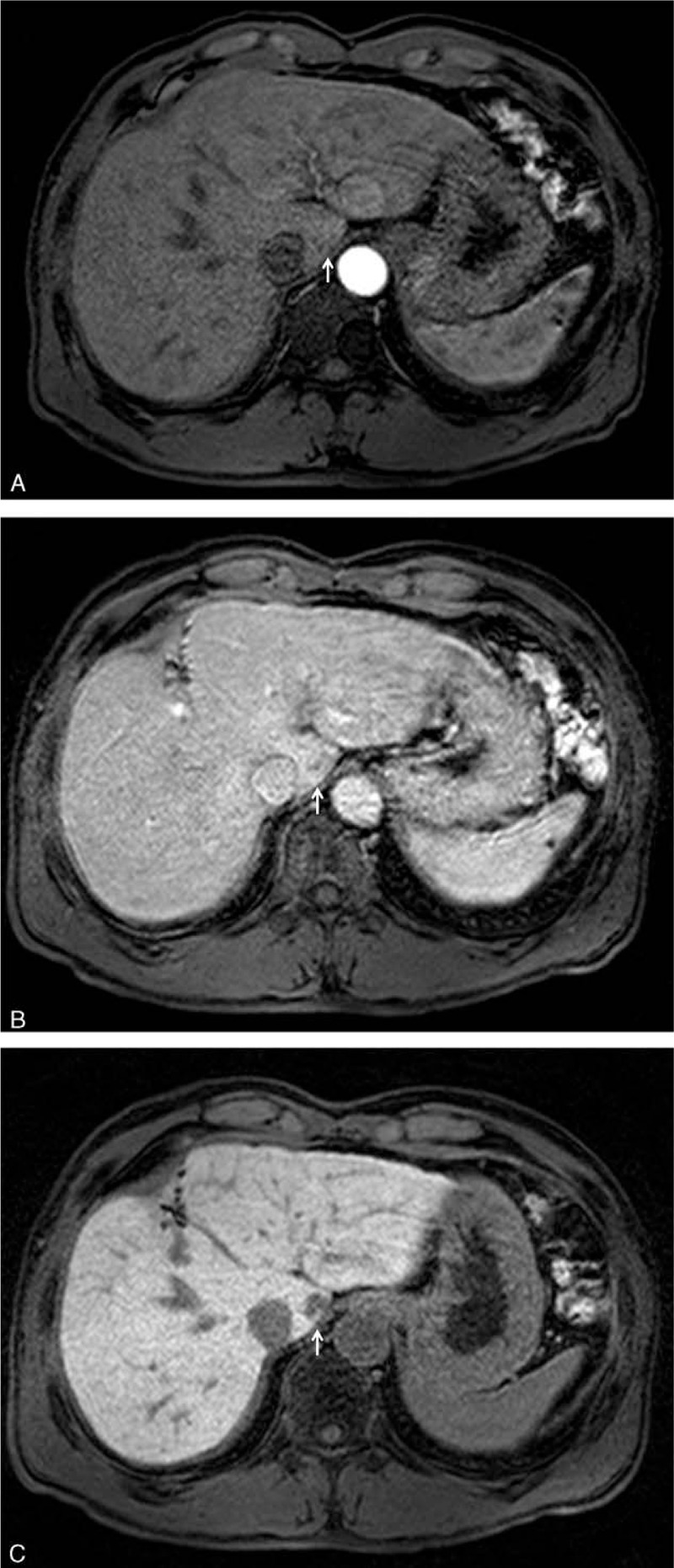
A 50-year-old man developed a 1.5-cm recurrent hepatocellular carcinoma 1 year after segmentectomy, which was detected with gadoxetic acid-enhanced magnetic resonance imaging but no lesion was found on the 3 months prior multidetector computed tomography (not shown). (A) Tumor enhanced on the arterial phase in segment 1 of the liver, appearing hyperintense to the background liver. (B) On the portal venous phase, tumor appeared hypointense to the background liver. (C) Tumor demonstrated no gadolinium ethoxybenzyl diethylentriamine pentaacetic acid uptake on the hepatobiliary phase.

The recurrent HCC detection rate of Gd-MRI and MDCT was 4.8% (9/180) and 4.3% (29/580), respectively, on the per test basis (*P* = 0.764). However, in the population with a follow-up period of ≥12 months, the recurrent HCC detection rate of Gd-MRI was higher than that of MDCT (4.3% [7/150] vs 1.5% [19/400]; *P* = 0.035) (Figs. 2 and 3).

### 3.2 Likelihood of recurrent HCC detection

**Figure 4:**
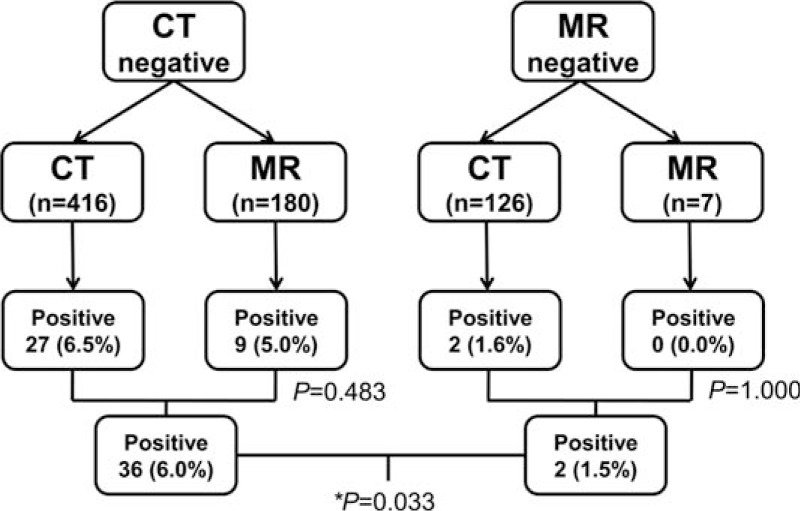
Likelihood of recurrent hepatocellular carcinoma detection in subsequent screening. CT = computed tomography, MR = magnetic resonance. ^∗^*P*-value <0.05.

Among the patients with negative results of surveillance with MDCT, recurrent HCC was detected in 6.5% (27/416) and 5.0% (9/180) by subsequent surveillance with MDCT and Gd-MRI, respectively (*P* = 0.483). However, among the patients with negative results of surveillance with Gd-MRI, recurrent HCC was detected only in 1.6% (2/126) and 0% (0/7) by subsequent surveillance with MDCT and Gd-MRI, respectively (*P* = 1.000). Overall, the likelihood of recurrent HCC detection in the subsequent screening after a negative result of Gd-MRI was lower than that of MDCT (1.5% [2/133] and 6.0% [36/596], respectively; *P* = 0.033) (Fig. 4).

### 3.3 Characteristics of the detected recurrent HCCs

The recurrent HCCs detected with Gd-MRI were smaller than that detected with MDCT (tumor size < 2 cm, 100% [9/9] vs 65.5% [19/29]; *P* = 0.040). The number of recurrent HCCs and additional treatment modality did not differ according to the screening modality (Table 2).

**Table 2:**
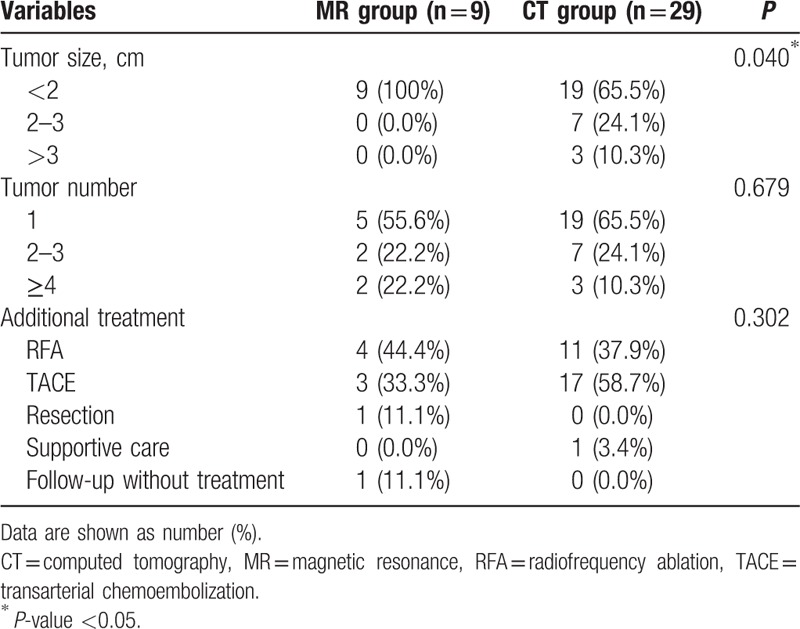
Comparison of detected recurrent tumor characteristics according to the surveillance test.

## 4 Discussion

Surgical resection, transplantation, and ablation are treatments that offer a high rate of complete response and, thus, potential for cure.^[2]^ However, long-term survival is still unsatisfactory due to the high incidence of recurrence. After resection, tumor recurrence rate, including recurrence due to dissemination and de novo tumors, exceeds 70% at 5 years.^[13,14]^ Accordingly, early detection and effective treatment of recurrent HCC is necessary to improve the long-term survival.^[4,15]^ Although a combined use of tumor markers and imaging studies is usually performed for postoperative surveillance, a consensus has not been established regarding the ideal imaging modality and interval. In this study, we investigated the additional value of Gd-MRI in surveillance for HCC after hepatectomy.

MDCT makes it possible to scan the entire liver multiple times during the artery-dominant phase, and increases the possibility of detecting arterial enhancement of tumor during the optimal phase. Indeed, MDCT has clearly improved the ability to detect the early staining of HCC compared with single detector CT.^[16]^ Recent improvements in MRI are also noticeable. Gd-MRI is of particular significance.^[17]^ Several studies have shown superiority of Gd-MRI compared with MDCT in various clinical settings.^[9,18–22]^ However, to date, there have been scant studies comparing Gd-MRI and MDCT in the surveillance setting after hepatectomy.^[6]^ Although Gd-MRI and MDCT were not performed at the same time, we could compare them by the HCC recurrence detection rates on the per test basis and detected tumor size.

A comprehensive review of recent studies on postoperative recurrence of HCC has shown a high incidence of recurrence in the first 1 to 2 years after resection, and defined early recurrence as recurrence within 1 year.^[23–25]^ Most early recurrences during follow-up are due to tumor dissemination and have a more aggressive biological pattern compared with primary tumors.^[13,26]^ On the other hand, the majority of late recurrences are attributable to new lesions and those patients with recurrence are due to de novo oncogenesis which can be expected to benefit from curative treatment.^[2,14]^ In our results, the recurrent HCC detection rates of Gd-MRI and MDCT did not differ in the overall patients. However, the recurrent HCC detection rate of Gd-MRI was higher than that of MDCT in the population with a follow-up period of ≥12 months. In other words, although early recurrences can be similarly detected with MDCT, Gd-MRI is more efficient in detecting late recurrences in earlier stages thereby increasing the potential for cure.

The likelihood of recurrent HCC detection differed in subsequent surveillance according to the prior surveillance modality. The recurrent HCC detection in subsequent surveillance after a negative result of Gd-MRI was less frequent than after a negative result of MDCT. In other words, a negative result of Gd-MRI may warrant a subsequent negative result for recurrent HCC with a higher probability than that of MDCT. Accordingly, Gd-MRI should supersede MDCT for safer and more reliable surveillance.

The goal of surveillance is identification of recurrent HCCs in its earliest stage when it is also the time for the highest possible likelihood of cure. Although the recurrent HCCs detected with Gd-MRI were smaller than those detected with MDCT, additional treatment modality did not differ according to the surveillance modality. However, the proportion of curative treatment for additional treatment is greater in the recurrent HCCs detected with Gd-MRI than that with MDCT (55.5% [5/9] vs 37.9% [11/29]), although it did not reach to the statistical significance. The ideal method to investigate the additional value of Gd-MRI would be a randomized controlled trial with a large sample size. However, the comparison between MDCT alone and alternating use of MDCT and Gd-MRI for surveillance imaging modality could be difficult because Gd-MRI has already been used for patients at a high risk of recurrence in practice. Accordingly, the authors investigated the utility of Gd-MRI by calculating the recurrent HCC detection rate and the likelihood of recurrent HCC detection in the cohort who received surveillance consisting of serum AFP measurement plus MDCT every 3 months along with annual Gd-MRI. Taken together, this study provides valuable information despite the limitations due to its retrospective design and small sample size.

In conclusion, our data suggest that Gd-MRI has an additional advantage of early detection of late recurrence. Therefore, surveillance with alternating MDCT and Gd-MRI may identify more recurrent HCC in an early stage when treatment has the highest possible likelihood of cure than with MDCT alone.
